# Enhanced Antitumor Effects of 5-Fluorouracil and a Vagal Nerve Blocker Loaded in a Thermosensitive Hydrogel for Localized Drug Delivery in Gastric Cancer

**DOI:** 10.3390/pharmaceutics18101224

**Published:** 2026-09-27

**Authors:** Cheng Wu, Xunjun Li, Yungeng Liu, Wu Zhong, Hongquan Liu, Jianzhong Yi, Shaoxiang Deng, Xiaoliang Lan, Chuanfa Fang, Tao Chen

**Affiliations:** 1 Department of Gastrointestinal and Hernia Surgery, Ganzhou People’s Hospital, Ganzhou 341000, China; 2 Department of General Surgery, Nanfang Hospital, Guangdong Provincial Engineering Technology Research Center of Minimally Invasive Surgery, Southern Medical University, No. 1838, North Guangzhou Avenue, Guangzhou 510515, China

**Keywords:** thermosensitive hydrogel, localized drug delivery, 5-fluorouracil, vagal nerve blocker, gastric cancer

## Abstract

**Background/Objectives:** Neural infiltration closely correlates with the progression and poor prognosis of gastrointestinal tumors. The vagus nerve regulates tumor cell proliferation and invasion through acetylcholine (ACh). Combining targeted neurotherapy with chemotherapy has recently emerged as a promising anticancer strategy. **Methods:** In this study, we designed a biodegradable temperature-sensitive hydrogel (PDLLA-PEG-PDLLA, PLEL) that co-delivers 5-fluorouracil (5-FU) and atropine (a vagal nerve blocker) for local gastric cancer chemotherapy. A key feature of this 5-FU + atropine/PLEL hydrogel system is its sol–gel phase transition in response to physiological temperature, enabling sustained drug release in vivo. **Results:** After intraperitoneal injection, this dual-drug-loaded hydrogel significantly reduced tumor growth in gastric cancer models, outperforming single-drug-loaded hydrogels and free 5-FU plus atropine combinations. This delivery system also reduced side effects. **Conclusions:** Therefore, this innovative dual-drug-loaded hydrogel shows strong potential for intratumoral gastric cancer chemotherapy and offers a clinically valuable option for postoperative management.

## 1. Introduction

Gastric cancer (GC) is a common digestive tract malignancy and ranks fifth in both incidence and mortality among all cancers [1]. More than 50% of patients with T3–T4 stage GC experience metastasis after surgical resection. Higher rates of peritoneal metastasis correlate with shorter survival [2]. Chemotherapy and radiotherapy play critical roles in preventing recurrence and metastasis in both operable and inoperable advanced GC. Nevertheless, about 50% of patients with advanced GC still die from recurrence and metastasis [3]. Thus, we urgently need new therapeutic strategies to reduce metastasis and recurrence in GC.

The tumor microenvironment (TME)—including both internal and external factors surrounding tumor cells—plays a key role in tumor development, growth, and metastasis. Nerves, as an essential component of the TME, promote tumor progression by interacting positively with other TME elements [4]. Targeting nerve fibers and signals has therefore become a novel and promising therapeutic approach. Since the early 20th century, researchers have paid increasing attention to tumor-associated nerve fibers, particularly perineural invasion (PNI) [5]. The incidence of PNI in the gastrointestinal tract ranges from 4% to 76% and is strongly associated with poor outcomes [6,7,8]. The location of GC correlates with nerve density, and combining denervation with chemotherapy can reduce tumor recurrence and improve prognosis [9].

The vagus nerve is closely linked to gastrointestinal tumor progression and prognosis, exerting diverse effects. It directly affects tumor cells through neurotransmitters, modulating their growth, proliferation, and metastasis [10]. Additionally, the vagus nerve influences the TME by regulating immune cells [11], affects gastrointestinal microbiota homeostasis via the brain–gut axis [12], and controls angiogenesis to support tumor growth [11].

The vagus nerve regulates tumor cell proliferation, invasion, and metastasis through ACh [13]. Acetylcholine receptors, including muscarinic (mAChRs) and nicotinic (nAChRs) subtypes, mediate these effects. Notably, the muscarinic type 3 receptor (M3R) plays a central role in gastrointestinal tumor progression. ACh comes from both neural and non-neural sources, including tumor and immune cells, and performs tumor-related functions [14]. Increased M3R expression in GC tissues associates with advanced tumor stage and lymph node metastasis, promoting cancer cell proliferation. Conversely, reducing M3R expression halts the cell cycle and triggers apoptosis [15]. Moreover, ACh activates EGFR signaling through M3R, leading to ERK1/2 and AKT phosphorylation. Inhibiting EGFR with AG1478 suppresses ACh-mediated proliferation [16]. This complex interplay among ACh, M3R, and EGFR highlights new opportunities for therapeutic intervention.

Although chemotherapy remains important in cancer treatment, it faces challenges such as severe side effects, drug resistance, and poor targeting [17]. Hydrogels have emerged as promising drug carriers that enable controlled loading, sustained release, and prolonged action. Their crosslinked aqueous structure reduces drug denaturation and aggregation, while their biocompatibility and biodegradability help mitigate side effects. Temperature- and pH-responsive hydrogels allow precise drug delivery, improving efficacy and reducing toxicity [18]. For example, Lee et al. developed a dual-responsive hydrogel that killed 80% of cancer cells within 48 h, showing strong potential to inhibit tumor growth [19]. Similarly, Chen et al. developed spermine-modified polymeric micelles with pH-sensitive drug release for targeted antitumor therapy, demonstrating that stimuli-responsive carriers can enhance drug accumulation at tumor sites while reducing systemic exposure [20]. Temperature-responsive hydrogels offer precise control, ease of use, sustained release, and compatibility with multimodal therapies, making them attractive for oncology [18]. They have therefore gained considerable attention as drug delivery platforms [21]. Injectable forms of these hydrogels gel rapidly at body temperature but remain soluble at room temperature, facilitating administration [22]. They can co-load multiple agents for targeted and sustained release [23,24,25,26,27]. For instance, Lv et al. used a DOX/cytokine-loaded hydrogel for enhanced chemo-immunotherapy [28], and Kim et al. developed a thermosensitive hydrogel for sustained release of nitric oxide donors and antibodies in cancer immunotherapy [29].

PDLLA-PEG-PDLLA, a biodegradable thermosensitive hydrogel, shows notable antitumor efficacy when co-delivering 5-FU and cisplatin for adjuvant GC chemotherapy [18]. Building on this, we developed a similar hydrogel system co-loaded with 5-FU and atropine for combined intraoperative GC treatment (Scheme 1). We easily prepared the 5-FU + atropine/PLEL hydrogel by physical mixing at room temperature, demonstrating high feasibility and operational flexibility. The hydrogel undergoes sol–gel transition at body temperature, allowing easy application over the tumor site, adjacent tissues, and abdominal cavity to target residual cancer cells. After gelation, the hydrogel provides sustained drug release, prolongs local drug retention, and suppresses postoperative recurrence and metastasis. We further evaluated the antitumor effect and reduced systemic toxicity of this system in a nude mouse model.

## 2. Materials and Methods

### 2.1. Samples and Patients

We conducted this study at Ganzhou People’s Hospital and collected gastric tissue samples from 28 patients undergoing radical gastrectomy for GC. The Ethics Committee of Ganzhou People’s Hospital approved the study and granted a full waiver of informed consent for the human tissue study (Approval No. PJB2025-316-01). The study was conducted in accordance with the ethical standards of the Declaration of Helsinki.

### 2.2. Cell Culture and Reagents

We obtained the GC cell lines MGC803 (RRID:CVCL_5334) and HGC27 (RRID:CVCL_1279) from the American Type Culture Collection (ATCC) in January 2022. After receipt, we expanded and cryopreserved the cell lines to create a master cell bank. We performed all experiments using low-passage cells (passages 5–15) from this bank. We routinely tested cell lines and confirmed they were free of mycoplasma contamination using a MycoAlert™ Mycoplasma Detection Kit (Lonza Rockland, Inc., Rockland, ME, USA, LT07). We cultured GC cells in RPMI-1640 medium (Gibco, Grand Island, NY, USA; Cat. No. C11875500BT) supplemented with 10% fetal bovine serum (FBS; Gibco, Grand Island, NY, USA) in a humidified incubator at 37 °C with 5% CO_2_. We purchased acetylcholine from Sigma-Aldrich (St. Louis, MO, USA), atropine from MedChemExpress (Monmouth Junction, NJ, USA; Cat. No. HY-B1205), and 5-fluorouracil from MedChemExpress (Monmouth Junction, NJ, USA; Cat. No. HY-90006).

### 2.3. CCK-8 Assay

We seeded cells into 96-well plates at 1000 cells per well and cultured them in RPMI 1640 medium with 10% FBS for 5 days. We assessed cell proliferation using the Cell Counting Kit-8 (CCK8; Beyotime, Shanghai, China; Cat. No. C0041). Briefly, we added 10 μL of CCK8 solution to each well, incubated the plates at 37 °C for 2 h, and then measured absorbance at 450 nm. We performed all experiments in triplicate.

### 2.4. Plate Clone Formation Assay

We plated cells in 6-well plates at 100 cells per well and cultured them in RPMI 1640 medium with 10% FBS. We allowed visible colonies to form over 12 days. Then we washed the colonies with phosphate-buffered saline (PBS), fixed them in 4% paraformaldehyde for 15 min, and stained them with 0.1% crystal violet for 15 min. We performed all experiments in triplicate.

### 2.5. Wound Healing Assay

We seeded cells into 6-well plates at 5 × 10^5^ cells per well and cultured them until they reached 90% confluence. We then replaced the complete medium with serum-free medium and used a sterile 100 μL pipette tip to scratch the cell monolayer. After further incubation for 48 h, we observed the wound gaps under an inverted light microscope at 100× magnification. We calculated the wound healing percentage using ImageJ 1.53 software: (healed wound area)/(initial wound area) × 100%.

### 2.6. Immunohistochemistry

We cut paraffin-embedded specimens into 4 μm slices. We dewaxed the slices with xylene, rehydrated them with alcohol, and then immersed them in EDTA antigen retrieval buffer, followed by microwave-assisted antigen retrieval. To inhibit endogenous peroxidase activity, we treated the slices with endogenous peroxidase block (Beyotime, Shanghai, China; Cat. No. P0100B) and then blocked nonspecific binding using immunostaining blocking solution (Beyotime, Shanghai, China; Cat. No. P0260). We incubated antibodies against M3R and Ki67 (Abcam, Cambridge, UK; Cat. Nos. ab314013, ab15580; diluted 1:500) with the slices overnight at 4 °C. After incubating with secondary antibodies, we stained the markers with DAB (ZSGBbio, Beijing, China; Cat. No. ZLI-9018) and counterstained the nuclei with hematoxylin. We captured images using an LSM980 (ZEISS) confocal microscope(Carl Zeiss Microscopy GmbH, Jena, Germany).

### 2.7. Hematoxylin and Eosin (H&E) Staining

We fixed samples in 10% formalin, cut them into 4–5 μm sections, and adjusted the pH. We stained sections sequentially with hematoxylin and eosin (Solarbio, Beijing, China; Cat. No. G1120). After staining, we dehydrated the sections through alcohol, cleared them in xylene, and mounted them using neutral resin. We captured images using the LSM980 confocal microscope (ZEISS).

### 2.8. Synthesis and Characterization of the Hydrogel

#### 2.8.1. Synthesis of 5-FU + Atropine/PLEL Hydrogel

We purchased the PDLLA-PEG-PDLLA copolymer from Xi’an Ruixi Biotechnology Co., Ltd. (Xi’an, China). Following the manufacturer’s instructions, we fully dissolved the copolymer in sterile water at a ratio of 1:4 at room temperature. After the hydrogel completely dissolved into a solution, we physically mixed 5-FU and atropine with the hydrogel solution at 25 °C to prepare the 5-FU + atropine/PLEL formulation. We kept the 5-FU concentration at 2 mg mL^−1^ and the atropine concentration at 0.1 mg mL^−1^.

#### 2.8.2. Characterization of Hydrogels

Polymer nuclear magnetic resonance (^1^H NMR): We dissolved a specific amount of the polymer in deuterochloroform (CDCl_3_) and analyzed the solution using a DMX 300 nuclear magnetic resonance spectrometer(Bruker BioSpin GmbH, Rheinstetten, Germany).

Gel permeation chromatography (GPC): We uniformly dissolved the samples in chloroform (CHCl_3_) for detection.

#### 2.8.3. Sol–Gel Phase Transition of PDLLA-PEG-PDLLA Hydrogel

We observed the thermosensitive sol–gel phase transition behavior of 5-FU + atropine/PLEL hydrogel using the tube inversion method. Specifically, we added 1 mL of the 5-FU + atropine/PLEL hydrogel sample to a 4 mL Eppendorf tube and heated it to 37 °C. We recorded changes in hydrogel state at room temperature and under heated conditions. We also recorded the phase transition behavior of the blank hydrogel at room temperature and 37 °C for comparison.

#### 2.8.4. Rheological Properties of PDLLA-PEG-PDLLA Hydrogels

We prepared PDLLA-PEG-PDLLA hydrogels by dissolving the copolymer in PBS at pH 7.4, with thorough stirring at room temperature to ensure complete dissolution. We evaluated the rheological properties of both blank and drug-loaded hydrogels using a NETZSCH Kinexus dynamic rheometer (NETZSCH-Gerätebau GmbH, Selb, Germany).

#### 2.8.5. Assessment of In Vivo Antitumor Efficacy of 5-FU + Atropine/PLEL Hydrogel

To establish an orthotopic tumor model in BALB/c nude mice, we injected approximately 40 μL of HGC27 GC cells (containing 2 × 10^6^ cells) into the subserosa of the anterior wall of the gastric antrum of 6-week-old nude mice. After successful model establishment, we randomly divided the tumor-bearing mice into six groups (n = 3 per group). We then intraperitoneally injected the mice with 200 μL of various solutions: normal saline (NS), PLEL solution, free 5-FU plus atropine (administered separately), atropine/PLEL hydrogel, 5-FU/PLEL hydrogel, and 5-FU + atropine/PLEL hydrogel. We maintained the doses of 5-FU [18] and atropine [30] at 20 mg/kg and 1 mg/kg, respectively, and administered them once every 6 days for a total of two doses. We recorded the body weight of each mouse every other day throughout the experiment.

On day 14 after treatment, we euthanized the mice and performed laparotomy to remove tumors and major organs. We fixed the resected tissues in 4% phosphate-buffered formalin for subsequent histological and immunohistochemical analyses. We calculated tumor volumes using the modified ellipsoidal formula: (length × width^2^)/2. All animal experiments followed the NIH Guide for the Care and Use of Laboratory Animals and were approved by the Ethics Committee of Ganzhou People’s Hospital (Approval No. PJD2025-045-01; date of approval: 1 September 2025).

### 2.9. Statistical Analysis

We present all data as mean ± standard deviation (SD). We performed statistical comparisons between groups using the *t*-test with GraphPad Prism 11.1.0 (GraphPad Software, San Diego, CA, USA). We considered statistical significance at * *p* < 0.05, ** *p* < 0.01, and *** *p* < 0.001.

## 3. Results

### 3.1. M3R Expression Is Upregulated in GC Tissues

We examined M3R expression levels in 28 GC tissues and their corresponding normal adjacent tissues. The results showed significantly higher M3R expression in GC tissues than in normal tissues (Figure 1A,B). This finding suggests a potential role for M3R in gastric carcinogenesis.

### 3.2. An Exogenous Muscarinic Receptor Agonist Enhances Proliferation and Migration of HGC27 and MGC803 GC Cells

To clarify the functional role of M3R in GC cells, we selected two human GC cell lines, HGC27 and MGC803, both known to express high M3R levels. We treated these cells with different concentrations of exogenous ACh to determine the optimal stimulatory concentration. Our data showed that 25 μM ACh produced the most pronounced effect on cell proliferation (Figure 2A,C). Using this concentration, we observed a time-dependent increase in the growth rate of both HGC27 and MGC803 cells in CCK8 assays (Figure 2B,D). Wound healing assays confirmed that 25 μM ACh stimulation accelerated wound closure, indicating enhanced migration (Figure 2E,F). Plate clone formation assays also showed that 25 μM ACh for 12 days significantly promoted the formation of larger and more numerous colonies in both cell lines (Figure 2G,H). Together, these results demonstrate that exogenous ACh increases proliferation and migration of HGC27 and MGC803 GC cells.

### 3.3. Preparation and Characterization of 5-FU + Atropine/PLEL Hydrogel

We thoroughly dissolved the PDLLA-PEG-PDLLA polymer in sterile water at room temperature at a polymer-to-water ratio of 1:4. We analyzed the polymer’s structural characteristics and molecular weight by ^1^H NMR spectroscopy (Figure 3C) and GPC (Figure 3B), respectively. The GPC analysis showed a unimodal molecular weight distribution with Mn = 5207 g/mol, Mw = 6764 g/mol, and PDI = 1.30 (Mw/Mn = 1.299). We then prepared the 5-FU + atropine/PLEL formulation by simple physical mixing of 5-FU and atropine with the PLEL micelle solution at 25 °C, keeping 5-FU at 2 mg mL^−1^ and atropine at 0.1 mg mL^−1^.

A critical property of PLEL hydrogels as drug carriers is their ability to undergo sol–gel transition at physiological temperatures. To evaluate this capability, we used the tube inversion method. The system remained a flowable sol at 25 °C, which facilitates drug loading and injection. As temperature increased, PLEL micelles enlarged, leading to aggregation and bridging, and finally forming a semi-solid hydrogel at 37 °C (Figure 3A). These findings indicate that PLEL hydrogels are injectable and can gel in vivo, making them promising drug carriers. Their sol–gel transition at physiological temperature not only enables easy administration but also ensures stable drug delivery after injection.

### 3.4. In Vivo Antitumor Efficacy and Toxicity of 5-FU + Atropine/PLEL Hydrogel

To further investigate the combined antitumor effects of 5-FU + atropine/PLEL hydrogel, we conducted animal experiments using a nude mouse orthotopic GC model. After tumor establishment, we intraperitoneally injected the hydrogels once every 6 days for two doses. We monitored body weights every 2 days until day 14, when we euthanized the mice for data collection (Figure 4A). Mice treated with drug-loaded hydrogels maintained stable body weights, indicating minimal systemic toxicity, in contrast to mice treated with free drug combinations (Figure 4B). Mice treated with NS, PLEL, or atropine/PLEL showed similar tumor volumes, whereas mice treated with 5-FU-containing hydrogels showed various degrees of tumor regression. Among these, the 5-FU + atropine/PLEL group had the strongest antitumor effect, with statistically significant differences compared with other groups (*p* < 0.05; Figure 4C,D).

To verify successful establishment of orthotopic gastric tumors, we performed H&E staining of mouse tumor tissue (Figure 5A). Importantly, Ki-67 staining revealed a much lower percentage of positive cells in the 5-FU + atropine/PLEL hydrogel group, indicating a substantial reduction in tumor cell proliferation (Figure 5B,C). Histological analysis confirmed these findings: mice treated with the hydrogel system showed minimal organ damage, whereas mice receiving free 5-FU and atropine displayed significant hepatocellular necrosis, disrupted splenic architecture, and glomerular atrophy (Figure 6). Thus, the 5-FU + atropine/PLEL hydrogel not only enhances the antitumor efficacy of combined GC chemotherapy but also effectively reduces systemic toxicity, making it a promising therapeutic option.

## 4. Discussion

Nerves are an important part of the tumor microenvironment and interact with multiple TME components to promote tumor development [31,32]. Our preliminary findings showed significantly worse outcomes in GC patients with PNI than in those without, highlighting the clinical relevance of this pathological feature [33]. Other studies agree that denervation can curb tumor progression, and when combined with chemotherapy, it reduces tumor recurrence and improves patient prognosis [9]. Currently, chemotherapy remains a cornerstone of cancer treatment; however, systemic toxicity and side effects from intravenous administration often limit its use [17]. Localized chemotherapy using drug-loaded biomaterials offers a promising clinical strategy to reduce systemic toxicity [34]. In this study, we developed a temperature-sensitive hydrogel delivery system loaded with 5-FU and atropine for local GC chemotherapy and evaluated its antitumor efficacy.

We observed higher M3R expression in GC tissues than in normal tissues. The vagus nerve releases ACh, which binds to M3R on target cells and modulates tumor progression. Previous studies have shown that ACh in tumor tissues promotes cancer cell proliferation and migration [35]. Our in vitro experiments confirmed that the exogenous M3R agonist ACh significantly increased proliferation and migration of HGC27 and MGC803 GC cell lines. These findings align with previous reports on M3R involvement in various cancers, emphasizing the broad relevance of M3R in cancer biology.

The vagus nerve influences tumor cell proliferation, migration, and invasion by releasing neurotransmitters such as ACh. Atropine, as a vagal nerve blocker, interrupts this signaling pathway and inhibits malignant tumor cell behavior [30]. Meanwhile, 5-FU, a classic antitumor drug, inhibits tumor growth by interfering with DNA synthesis and cell division [36]. The combination of these two agents both blocks tumor cell growth signals and directly disrupts proliferative capacity, producing a potent synergistic antitumor effect. In the 5-FU + atropine/PLEL hydrogel treatment group, the marked reduction in Ki-67-positive cells and its downregulation indicate effective suppression of tumor growth. This finding further supports targeting M3R together with chemotherapy for GC treatment.

Hydrogels serve as excellent drug delivery systems and are widely used for antitumor therapy across various tumor types. Their thermosensitive properties and sustained drug release ensure prolonged drug action at tumor sites, thereby improving therapeutic efficacy. Beyond drug delivery, hydrogels have also been explored as intelligent biosensing platforms. For instance, Yao et al. recently developed size-selected DNA hydrogels for efficient intracellular delivery and sensitive mRNA imaging, highlighting the versatility of hydrogel-based systems in biomedical applications [37]. In our study, the 5-FU + atropine/PLEL group showed superior antitumor effects compared with the free 5-FU + atropine group. Moreover, intraperitoneal injection of the drug-loaded hydrogels achieved localized and precise drug release at the tumor site, minimizing systemic toxicity and adverse effects [38]. We observed that nearly all major organs in the hydrogel-treated groups showed histological morphologies similar to those of mice in the NS control group, indicating low systemic toxicity. In contrast, mice treated with free 5-FU showed obvious hepatocyte necrosis, indistinct boundaries between splenic cortex and medulla, and glomerular atrophy. Therefore, the 5-FU + atropine/PLEL hydrogel not only improves the antitumor efficacy of combined GC chemotherapy but also effectively reduces systemic toxicity.

We acknowledge several limitations in this study. Although we have observed that adding a vagal nerve blocker enhances the antitumor efficacy of 5-FU, the precise mechanism underlying the antitumor effect after blocking acetylcholine receptors requires further investigation.

## 5. Conclusions

Our study demonstrates that M3R is a promising therapeutic target in GC. Inhibiting M3R together with chemotherapy using 5-FU/PLEL hydrogels represents a novel and effective treatment strategy. The local and sustained release of drugs from the hydrogel minimizes systemic toxicity while maximizing antitumor effects. Future studies should focus on optimizing the hydrogel formulation and exploring its clinical potential in larger cohorts of GC patients.

## Figures and Tables

**Figure 1:**
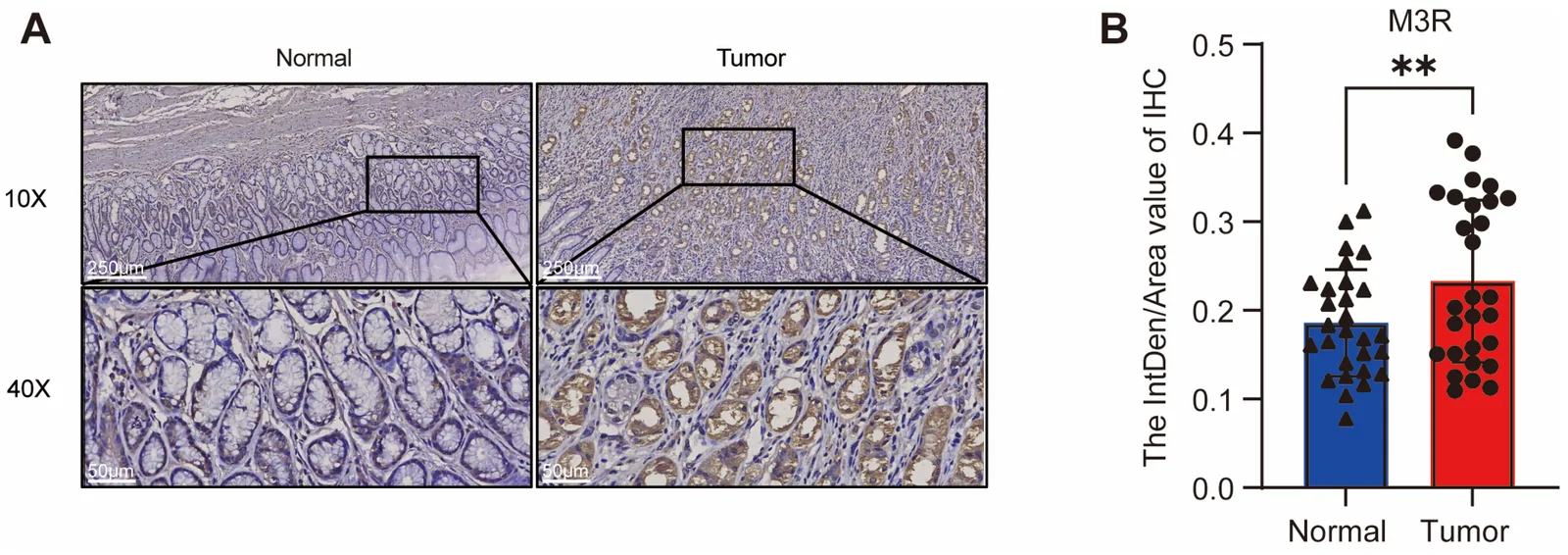
M3R is upregulated in GC tissues. (**A**) Representative IHC images of M3R in human GC tissues and adjacent noncancerous tissues. (**B**) Relative expression of M3R in 28 pairs of GC and adjacent normal tissues (paired *t*-test). Blue bars, adjacent normal tissues; red bars, GC tissues. ** *p* < 0.01.

**Figure 2:**
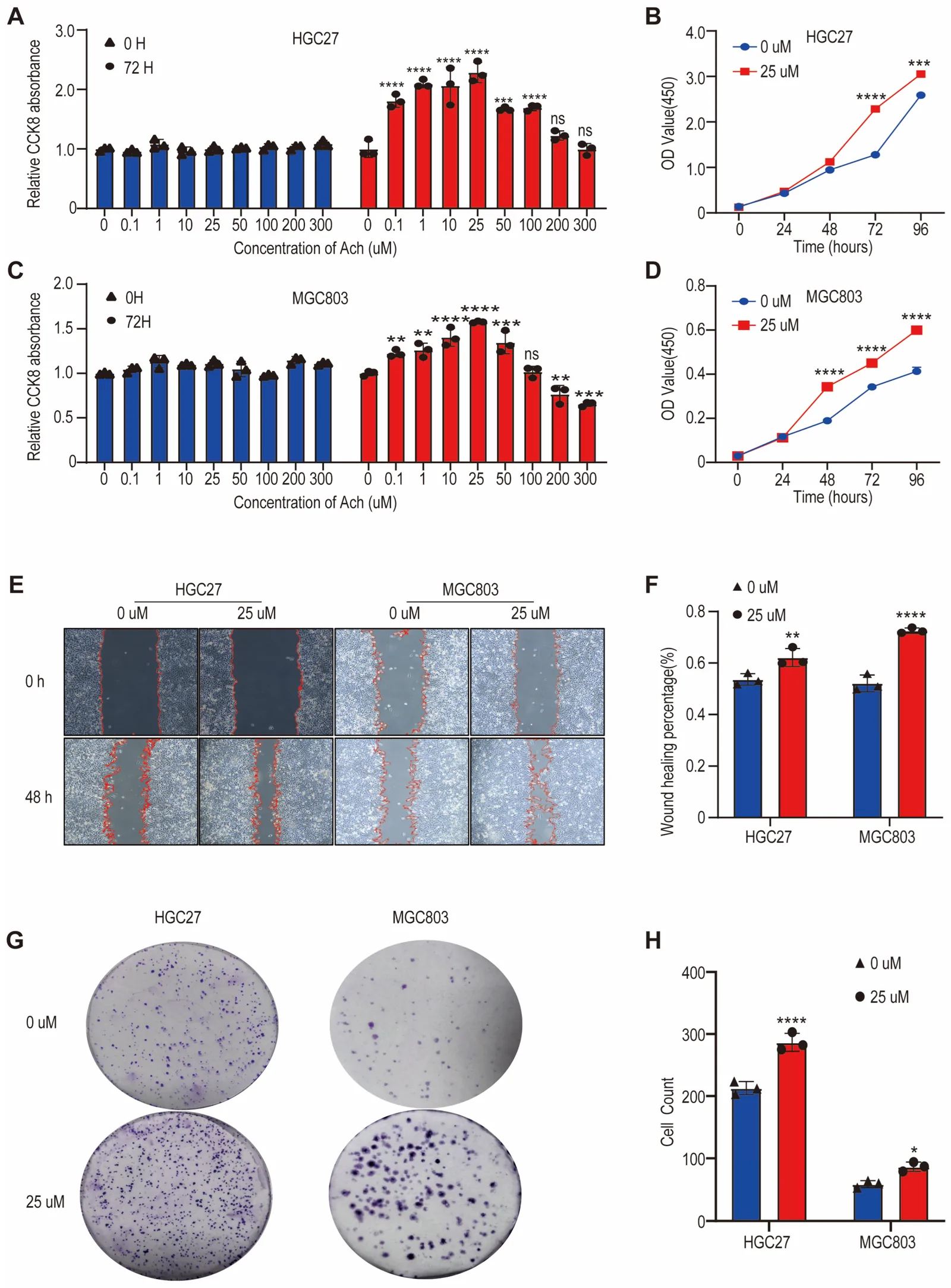
Effect of ACh on proliferation and migration of MGC803 and HGC27 cells. (**A**,**C**) HGC27 and MGC803 cells incubated with different ACh concentrations (0–300 μM) for 3 days; proliferation evaluated by CCK8 assay. (**B**,**D**) HGC27 and MGC803 cells incubated with 25 μM ACh for different days; proliferation evaluated by CCK8 assay. (**E**,**F**) Wound healing assay of HGC27 and MGC803 cells induced by 25 μM ACh. (**G**,**H**) Colony forming capacity of HGC27 and MGC803 cells induced by 25 μM ACh. In (**A**,**C**), blue bars, 0 h; red bars, 72 h. In (**B**,**D**), blue lines, 0 μM ACh; red lines, 25 μM ACh. In (**E**), red lines indicate wound edges. In (**F**,**H**), blue bars, 0 μM ACh; red bars, 25 μM ACh. ns, not significant. * *p* < 0.05, ** *p* < 0.01, *** *p* < 0.001, **** *p* < 0.0001.

**Figure 3:**
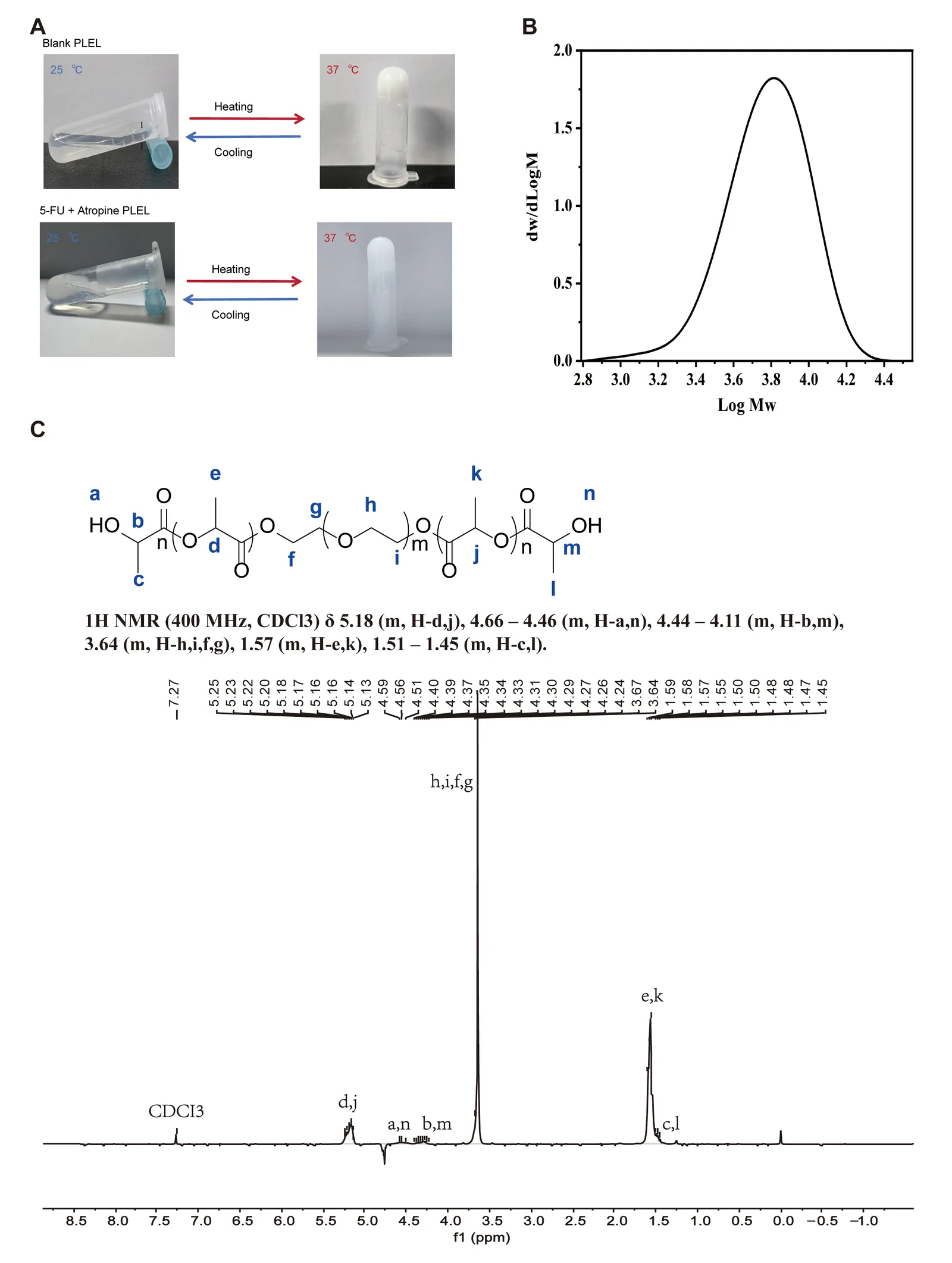
Characterization of PDLLA-PEG-PDLLA hydrogels. (**A**) Reversible sol–gel phase transition of blank PLEL hydrogel and 5-FU + atropine/PLEL hydrogel (polymer concentration: 20 wt%; 5-FU: 2 mg/mL; atropine: 0.1 mg/mL) between 25 and 37 °C. (**B**) Molecular weight distribution of PDLLA-PEG-PDLLA triblock copolymer. (**C**) ^1^H-NMR spectrum of PDLLA-PEG-PDLLA copolymer. The letters a–n in (**C**) indicate the proton assignments corresponding to the chemical structure of the copolymer; the detailed peak assignments are listed in the text.

**Figure 4:**
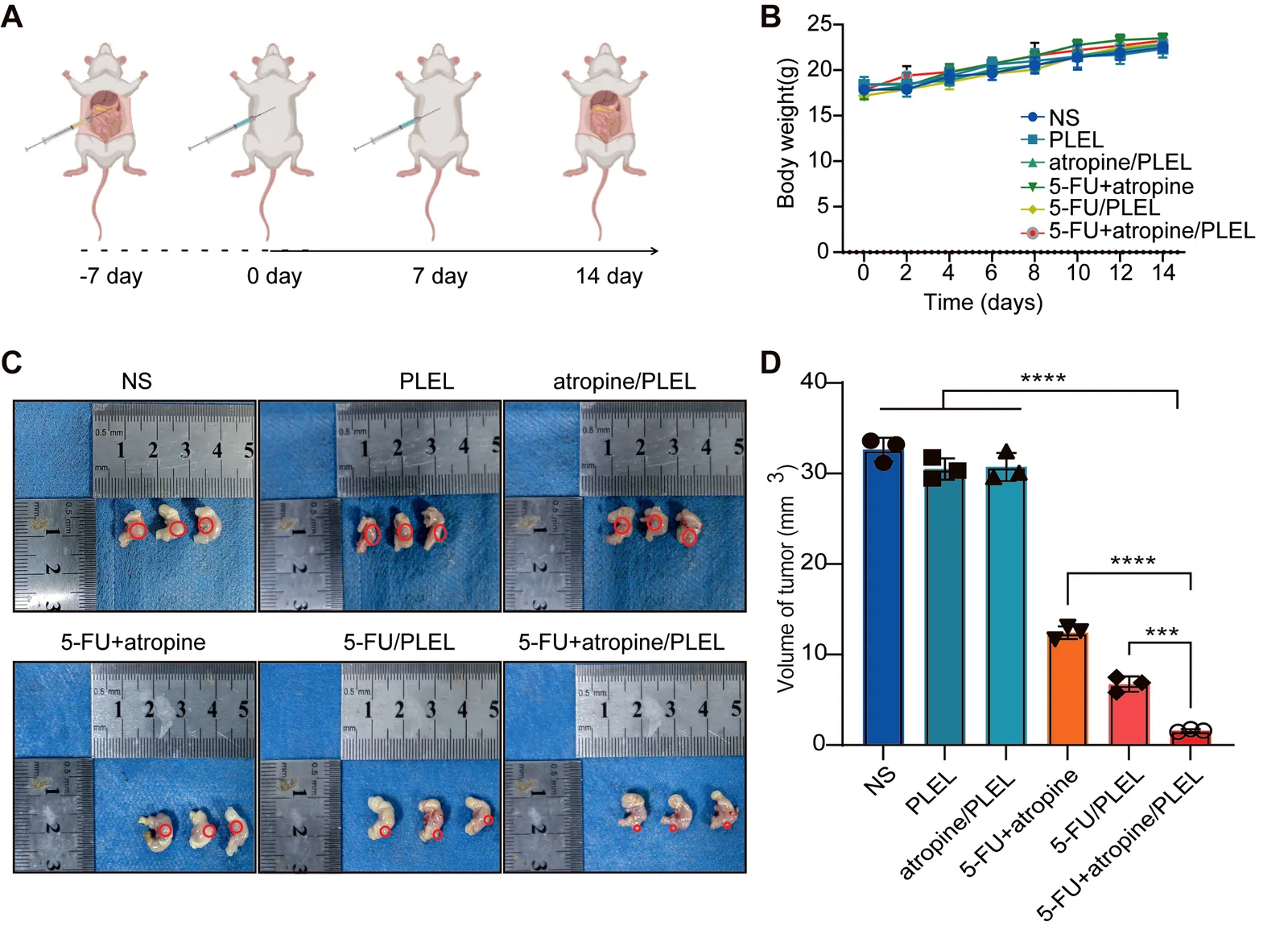
In vivo antitumor effect of different treatments in GC models. (**A**) Schematic diagram of GC model establishment in nude mice. (**B**) Body weight changes after drug administration. Data are mean ± SD (n = 3). (**C**) Tumor images after different treatments. Red circles indicate tumor tissues. (**D**) Tumor size in different groups. Data are mean ± SD (n = 3). In (**B**,**D**), colors correspond to the treatment groups as indicated: NS, PLEL, atropine/PLEL, 5-FU + atropine, 5-FU/PLEL, and 5-FU + atropine/PLEL. *** *p* < 0.001, **** *p* < 0.0001.

**Figure 5:**
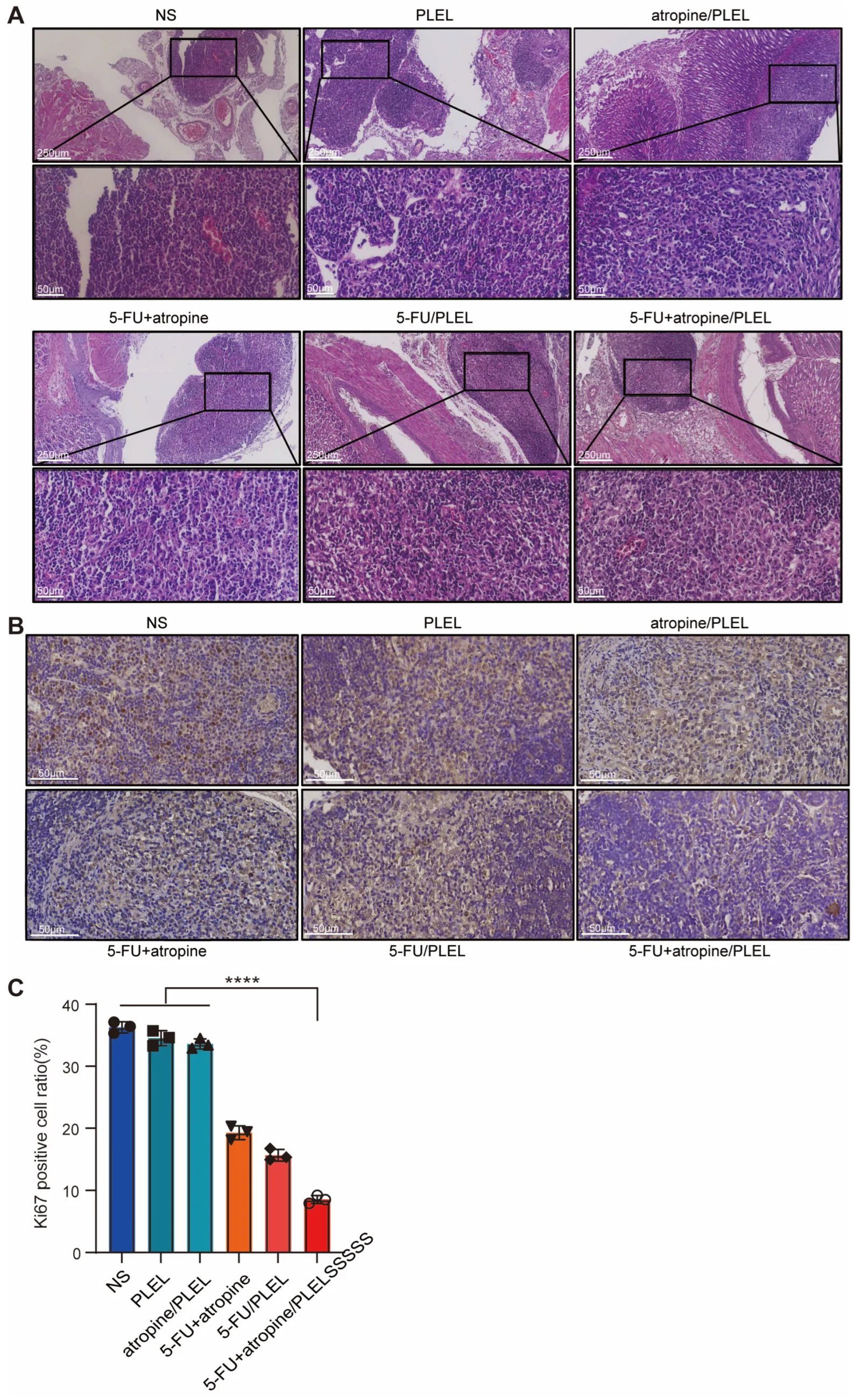
Tumorigenesis validation and antitumor efficacy of different therapeutic regimens in mice. (**A**) H&E staining images of mouse tumors (scale bar: 250 μm) and magnification (scale bar: 50 μm). (**B**) Ki-67 assay images of mouse tumor tissues from different treatments (scale bar: 50 μm). (**C**) Percentage of Ki67-positive cells after treatment. Data are mean ± SD (n = 3). In (**C**), colors correspond to the treatment groups as in Figure 4. **** *p* < 0.0001.

**Figure 6:**
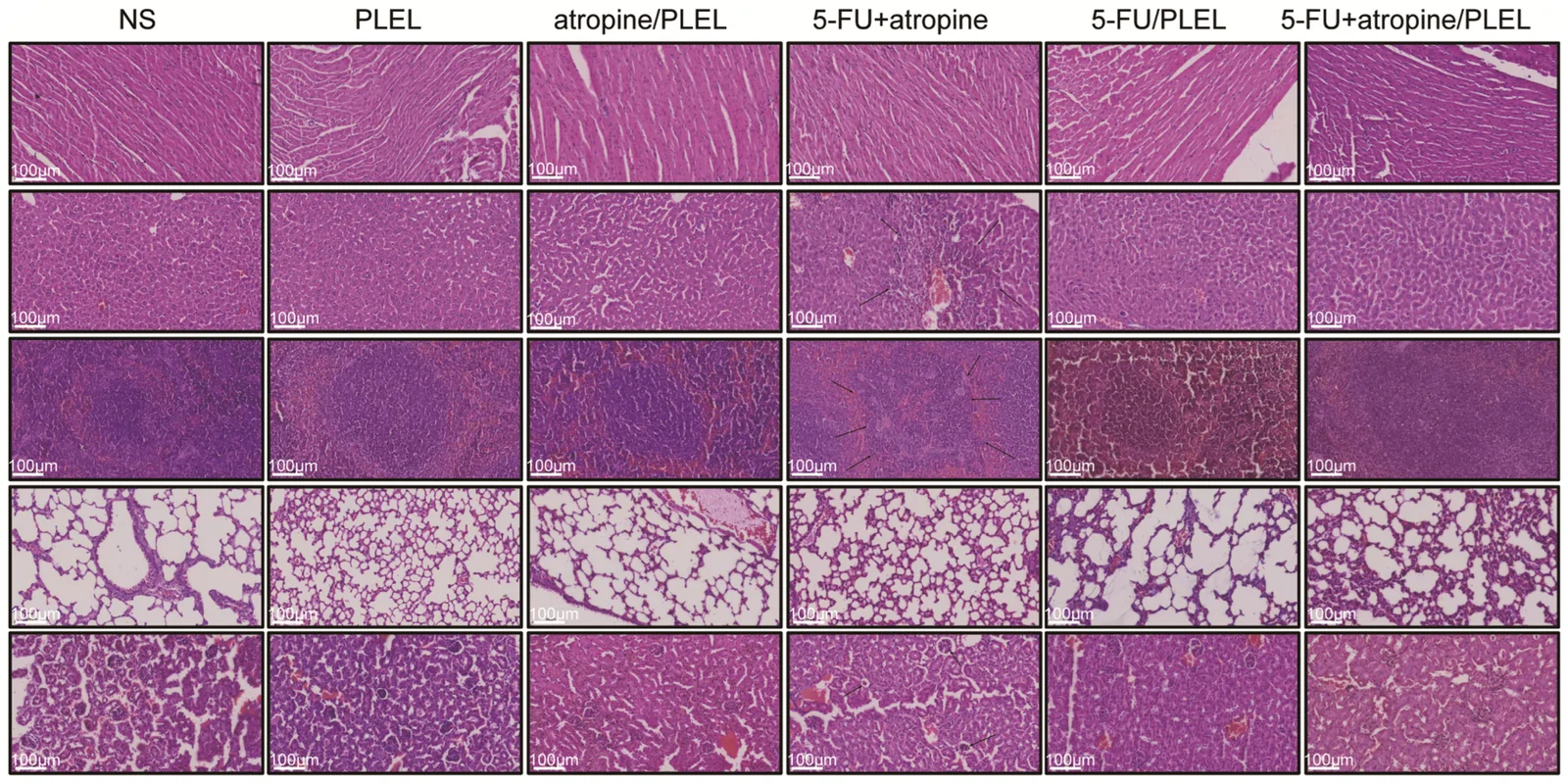
Safety assessment of the dual-drug loaded hydrogel antitumor regimen. H&E staining of major organs after different treatments. Black arrows indicate tissue damage.

**Scheme 1:**
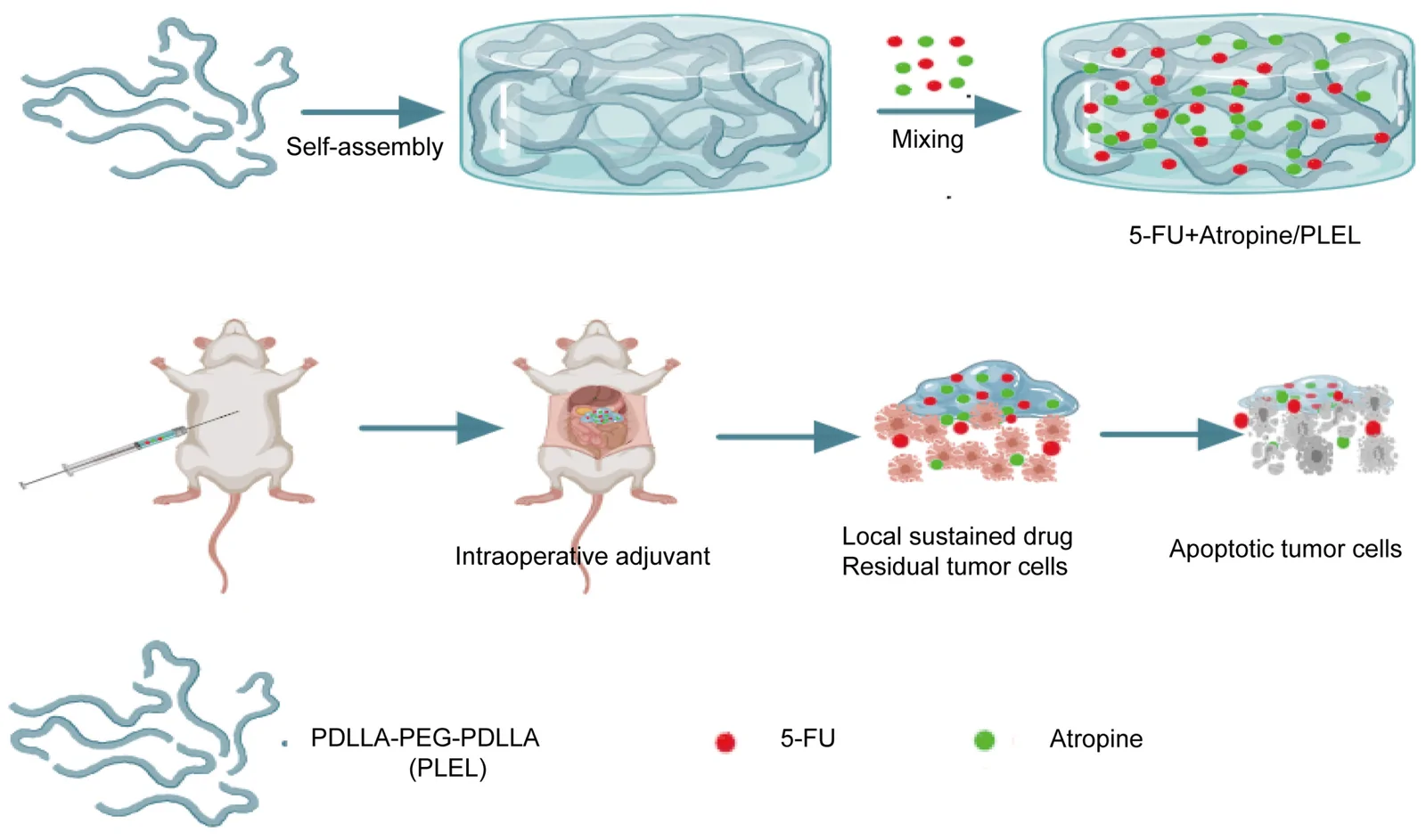
Schematic illustration of sustained co-delivery of 5-FU and atropine using a biodegradable thermosensitive hydrogel to enhance the antitumor effect of local drug administration in gastric cancer.

## Data Availability

The original contributions presented in the study are included in the article, further inquiries can be directed to the corresponding author.
